# Specific and quantitative detection of Human polyomaviruses BKPyV and JCPyV in the healthy Pakistani population

**DOI:** 10.1186/s12985-017-0752-2

**Published:** 2017-04-24

**Authors:** Iqra Hussain, Fareeda Tasneem, Muhammed Umer, Ayesha Pervaiz, Muslim Raza, Muhammad Imran Arshad, Naveed Shahzad

**Affiliations:** 1 School of Biological Sciences, University of the Punjab, Lahore, Pakistan; 2 Department of Zoology, University of the Punjab, Lahore, Pakistan; 3 National Institute for Biotechnology & Genetic Engineering, Faisalabad, Pakistan; 4 Department of Mathematics and Statistics, Virtual University of Pakistan, Lahore, Pakistan; 5 Institute of Microbiology, University of Agriculture, Faisalabad, Pakistan

**Keywords:** BKPyV, JCPyV, qPCR, Healthy blood, Pakistan

## Abstract

**Background:**

The BK Polyomavirus (BKPyV) and JC polyomavirus (JCPyV) infections are widespread in human population and have been associated with severe kidney and brain disorders, respectively. The viruses remain latent primarily in reno-urinary tract, reactivating only in case of a compromised immune system. The seroepidemiology and molecular prevalence of BKPyV and JCPyV have been widely studied both in healthy and immunocompromised patients worldwide. However, data regarding the prevalence of these viruses in the immunocompetent or apparently healthy Pakistani population is lacking. Herein, we present the first ever report on quantitative prevalence of BKPyV and JCPyV in the peripheral blood of a randomly selected cohort of healthy Pakistani population.

**Methods:**

A total of 266 whole blood samples were examined. The subjects were divided into three age groups: ≤ 25 years (young), 26–50 years (middle) and ≥ 51 years (elder). Absolute real time PCR assay was designed to quantify the BKPyV and JCPyV viral copy numbers in the range of 10^6^ to 10^0^ copies/mL.

**Results:**

Overall, BKPyV was detected in 27.1% (72/266) individuals while JCPyV in 11.6% (31/266) indicating significant difference (*p* < 0.005) in the distribution of these two viruses. The prevalence of BKPyV significantly decreased from 51% (49/96) in young age group to 8.2% (7/85) in eldest age group. Whereas, JCPyV positivity rate slightly increased from 8.3% (8/96) in young age group to 11.8% (10/85) in elder age group. The median viral load was calculated as 6.2 log and 3.38 log copies/mL of blood for BKPyV and JCPyV, respectively. Notably, no significant difference in viral load of either of the subtypes was found between different age groups.

**Conclusion:**

The current study provides an important baseline data on the prevalence and viral load of circulating BKPyV and JCPyV in Pakistani population. The prevalence and viral load of BKPyV was comparatively higher than JCPyV. The prevalence of BKPyV significantly decreased with increase in age while JCPyV positivity rate slightly increased with increasing age. Viral load of both BKPyV and JCPyV was not correlated with the individual ages.

**Electronic supplementary material:**

The online version of this article (doi:10.1186/s12985-017-0752-2) contains supplementary material, which is available to authorized users.

## Background

The first two human polyomaviruses BK polyomavirus (BKPyV) and JC polyomavirus (JCPyV) were described in 1971 by Gardner et al. and Padget et al. in two separate reports respectively [1, 2]. Since then there has been considerable research to unravel the molecular structure, pathogenesis, and biology of various polyomaviruses. With the recent discoveries of many new species, there has been a heightened focus on polyomavirus research. Primary BKPyV and JCPyV infections are usually asymptomatic or tend to be mild respiratory or gastrointestinal tract infection [3, 4]. Immunocompetent individuals can be infected with these two viruses at early ages, where the viruses may remain latent in various body organs like the reno-urinary tract and lymphoid tissues [5]. The destabilization of immune system due to the organ transplant or other immunocompromising conditions like severe acquired immunodeficiency (AIDS) may lead to the reactivation of BKPyV and JCPyV probably due to the deficiency of cytotoxic and helper T cells that are no longer available to keep a check on the virions [6]. The reactivation of BKPyV and JCPyV in immunocompromised individuals has been linked with several human malignancies. The BKPyV is reported to cause hemorrhagic cystitis and polyomavirus associated nephropathy (PVAN) while JCPyV is linked with Progressive Multifocal Leukoencephalopathy (PML) [7].

The BKPyV and JCPyV have circular double stranded DNA genome of approximately 5 kb which is organized into three regions named as the early, the late and the middle regions [8, 9]. The early region of BKPyV transcribes three transforming proteins called T antigens; LT antigen, ST antigen, and Truncated T antigen, whereas JCPyV early region encodes five T antigens; LT antigen, ST antigen, T’135, T’136 and T’165 antigens [10, 11]. The late region of both BKPyV and JCPyV encodes four proteins VP1, VP2, VP3 and agnoprotein. Likewise, the non-coding control region (NCCR) of both viruses is arranged in between early and late region having origin of replication [11].

The upper urinary tract is primary site of productive and replicative BKPyV infection. However, antigens of BKPyV can also be detected in many other tissues, almost in every cell type of respiratory tract, skin, bone, brain, colon and in the blood [12, 13]. On the other hand, JCPyV has a restricted tissue tropism range due to interaction with specific host cell surface α 2, 6-linked sialic acid receptors [14]. Oligodendrocytes and astrocytes of brain, kidney cells, lung epithelial cells and B lymphocytes display this receptor and are the sites of primary infection and reactivation [15]. Both of these viruses have also been demonstrated to remain latent in the peripheral mononuclear cells (PBMCs) particularly lymphocytes of healthy immunocompetent individuals [16–19]. The presence of BKPyV in PBMCs is thought to be associated with the ability of virus to manipulate host immune system so as to facilitate its spread from site of primary infection to the site of persistence [20]. However, it is not clear whether JCPyV remain latent in CNS and reactivates upon immunosuppression or it persists in blood from where it can transport to CNS after reactivation [21, 22].

Different seroprevalence studies as indicator of primary infection conducted around the globe have revealed that antibodies against BKPyV and JCPyV are present in 60–80% of healthy individuals [23]. Despite being ubiquitous, PyV infection in immunocompetent individuals does not do any harm to them that is of clinical significance. However, estimation of PyV infection in healthy population is of importance as it can not only provide the baseline data for risk assessment of active infection but can also shed light on the molecular epidemiology of various virus species in different geographical areas. Diverse molecular techniques have been employed over the last few years for rapid and specific detection of BKPyV and JCPyV DNA in immunocompetent individuals. However, the utilization of Real-time PCR (qPCR) remained on the top because of its superior sensitivity and specificity towards the detection as well as measurements of the PyVs [24]. Nevertheless, molecular based studies have manifested contrasting results about the distribution of BKPyV and JCPyV in different geographical regions of the world [25]. Unfortunately, to the best of our knowledge, not even a single study about the prevalence and quantification of BKPyV and JCPyV in Pakistani population has so far been conducted. Keeping in view the high seropositivity rate of BKPyV and JCPyV across the world it is imperative to study the molecular epidemiology of these viruses in Pakistan. Herein we present the first ever report on quantitative prevalence of BKPyV and JCPyV in apparently healthy Pakistani population.

## Methods

### Blood samples and subjects

The study was approved by the Ethics Committee of School of Biological Sciences, University of the Punjab, Lahore, Pakistan. A total of 266 individuals (males = 183, females = 83) from different localities of Punjab province, aged 10–80 years, were selected randomly. All participating individuals were asked for their consent on a written proforma. All the selected donors were negative for HBV, HCV and HIV infection. The population under study was divided into three age groups; ≤ 25 years (young age group), 26–50 years (middle age group) and > 51 years (older age group) in order to check the variations in the prevalence of these viruses in different ages. The young age group comprised of 96 individuals while the rest of two groups included 85 individuals each. A total of 5 mL peripheral blood was collected from each individual in vacutainers having EDTA (Becton Dickinson, USA) and stored at −20 °C until further testing since long term storage of whole blood at −20 °C does not affect the quality of DNA [26].

### DNA extraction and quality assay

The DNA was extracted from 1 mL whole blood using salt precipitation method described elsewhere [27]. Briefly, 1 mL of whole blood from each sample was mixed with 1 mL Buffer A (0.32 M sucrose, 10 mMTrisCl, 5 mM MgCl_2_ and 0.75% Triton-X-100, pH 7.6) and 1 mL of cold deionized water in sterile tubes followed by incubation on ice for 2–3 min. Tubes were centrifuged at 3500 rpm for 15 min at room temperature. Obtained pellets were resuspended in 1 mL of Buffer A and 3 mL of water and centrifuged again at above mentioned conditions. Following centrifugation, pellets were resuspended in 3 mL of Buffer B (20 mMTris-HCl, 4 mM Na_2_EDTA and 100 mM NaCl, pH 7.4) and 500 μL of 10% SDS (10gm SDS in 100 mL water) by gentle vortexing. Mixtures were incubated at 55 °C for 2 h by adding 10 μL of refrigerated proteinase K solution (20 mg/mL). Following incubation, 3 mL of 5 M NaCl was added and samples were centrifuged at 4500 rpm for 20 min. The supernatants were collected into other tubes carefully and equal volume of ice cold isopropanol was added. Pellets were collected by following the centrifugion at 4500 rpm for 20 min and washed with 70% ethanol. Finally pellets were suspended in 100 μL of DNase/RNase free water and stored at −20 °C.

The quantity of DNA was measured by nanodrop (DeNovix DS-11 Spectrophotometer) and 260\280 ratios were confirmed. Quality of extracted DNA was assessed by gel electrophoresis. β-globin polymerase chain reaction was performed with all DNA specimens to check the suitability of the samples for PCR analysis and the absence of inhibitors in the reaction. The primers used to amplify a 268 bps region of β–globin genes were; forward primer 5′-CAACTTCATCCACGTTCACC-3′ and reverse primer 5′-GAAGAGCCAAGGACAGGTAC-3′ [28]. The reaction mixture of 25 μl was prepared and the amplification profile was set in thermocycler (Eppendorf) for 10 min at 95 °C for activation of Taq DNA polymerase followed by 30 cycles of denaturation (95 °C for 30 s), annealing (59 °C for 30 s) and extension (72 °C for 30 s) and final extension at 72 for 10 mints.

### Positive controls and standard curves for BKPyV and JCPyV

The *LT antigens* of BKPyV (Dunlop strain) and JCPyV (MAD1 strain) cloned in pcDNA3 expression vector, were a kind gifted from Dr. Tommasino, International Agency for Research on Cancer, Lyon, France. These plasmids were used as positive controls for BKPyV and JCPyV standard curves preparation. The standard curves were plotted for each virus by using 10-fold serial dilutions of positive control plasmids as described elsewhere [29]. Briefly, the mass of a single plasmid molecule was calculated first by using the following formula,

$$ \mathrm{m} = \left[\mathrm{n}\right]\left[1.096\times {10}^{\hbox{-} 21}\mathrm{g}/\mathrm{bp}\right]\kern1.25em \mathrm{Where}:\ \mathrm{n}=\mathrm{Plasmid}\ \mathrm{size}\left(\mathrm{bp}\right)\ \mathrm{and}\ \mathrm{m}=\mathrm{mass} $$

The mass of plasmid needed for the copy number of interest was calculated as below,

$$ \mathrm{Mass}\ \mathrm{o}\mathrm{f}\ \mathrm{plasmid}\ \mathrm{D}\mathrm{N}\mathrm{A}\ \mathrm{needed} = \mathrm{Copy}\ \mathrm{N}\mathrm{o}.\ \mathrm{o}\mathrm{f}\ \mathrm{interest} \times \mathrm{mass}\ \mathrm{o}\mathrm{f}\ \mathrm{single}\ \mathrm{plasmid} $$

The concentration of plasmid DNA required to prepare the copy number of interest was calculated by dividing the mass plasmid DNA needed by the vol. to be pipetted into each reaction. The 10 fold serial dilutions from maximum copy number dilution (10^6^) to lowest copy number dilution (10^0^) were prepared. The input copy number range was selected from 10^6^ to 10^0^ in a way that 5 μL of each standard dilution used in 20 μL PCR reaction contained the desired copy number. These standard controlled dilutions were stored at −20 °C as aliquots until used.

### *LT antigen* primers and cross reactivity

The full length *LT antigen* sequences of BKPyV (Dunlop strain) and JCPyV (MAD1 strain) were retrieved from GenBank from accession code NC_001538 and NC_001699, respectively. These sequences were used to design primers for the amplification of the specific target gene *Large T antigen* of BKPyV and JCPyV by using primer quest bioinformatics tool. A forward primer 5′-AATATTATGCCCAGCACACATG-‘3 and reverse primer 5′-CTTTCCCTCTGATCTACACCAG-‘3 was used to amplify a 155 bps region of *LT antigen* of BKPyV. Likewise, a pair of forward primer 5′-AGAGTGTTGGGATCCTGTGTTTT-‘3 and reverse primer 5′-TTGCAGGGCATTTTGTTTTTTAC-‘3 were used to amplify a 177 bps region of *LT antigen* of JCPyV. In order to check the cross reactivity of BKPyV and JCPyV primers, each primer set was subjected to standard PCR to amplify *LT antigen* target gene of both viruses by employing irrespective targets. Amplification profile was set as 10 min at 95 °C followed by 35 cycles of denaturation (95 °C for 30 s), annealing (57 °C for 1 min) and extension (72 °C for 1 min) and final extension at °C for 10 min.

### Real time PCR for the quantification of BKPyV and JCPyV DNA

Fluorogenic quantitative real time PCR was carried out on PikoReal real time detection system (Thermo Fisher Scientific) to detect and quantify BKPyV and JCPyV viral loads. For each 20 μL PCR reaction mixture, 10 μL of maxima SYBR green qPCR 2X master mix (Thermo Fisher Scientific), 0.3 pmol of each BKPyV *LT* forward and reverse primer, 3.8 μL sterile water and 5 μL of 6 ng/μL DNA samples was used. For each sample, the real time PCR was performed in triplicate in 96 well plates. Each plate contained blood DNA samples, BKPyV standard controls (10^6^ to 10^0^ copy numbers) as well as water and empty pcDNA3 as negative control. Thermal cycling was initiated with a denaturation step of 95 °C for 10 min. It was followed by 50 cycles of 95 °C for 30 s and 57 °C for 30 s, 72 °C for 15 s and final extension at 72 °C for 5 min. Melt curve analysis was included in real time PCR protocol. Following conditions were used for melt curve 55 °C to 95 °C: Increment 1 °C for 1 min. Same protocol and reaction set up was repeated for JCPyV detection and quantification. Melt curve, melt peak and other data analysis was performed with PikoReal 2.2 software. The standard amplification curve of known quantity of DNA (*LT*-BKPyV-pcDNA3 and *LT*-JCPyV-pcDNA3) was used to determine the unknown amount of DNA in the test samples. The lower limit for the detection of BKPyV and JCPyV was 10^0^ and 10^1^ copies per reaction respectively. Some amplified PCR products were also checked and confirmed by agarose gel electrophoresis.

### Statistical analysis

The statistical analysis was carried out using SPSS software package version 19 (IBM, NY). The data is presented as the Mean ± SEM. The standard two sample *t*-test was used to test differences between the means. One way ANOVA is applied for testing of equivalence of the three age group averages, whereas the Chi-square test is applied for testing the equality of proportion. The *p*-values less than 0.05 were considered statistically significant and vice versa. MS EXCEL 2010 and SPSS 19 softwares were used for computational purpose.

## Results

### Demographic characteristics of the studied human population

A total of 266 blood samples of healthy individuals from different districts of Punjab region Pakistan, were collected for this study. Out of these, 68.8% (*n* = 183) individuals were male and 31.2% (*n* = 83) were females. These samples were divided into three age groups. The number of individuals in each age group along with mean and median ages is shown in Table 1.

**Table 1:** Demographics of the study participants

Age Groups	Ages (Years)	Participants n (%)	Mean ± S.D Age (Years)	Median ± S.D Age (Years)
Young age group	≤25	96 (36.2%)	21 ± 2.17	20 ± 2.40
Middle age group	26–50	85 (31.9%)	36.21 ± 7.70	36 ± 7.47
Old age group	≥51	85 (31.9%)	63.24 ± 8.93	61 ± 8.81

### DNA isolation and evaluation of BKPyV and JCPyV *LT* primers for cross reactivity

Genomic DNA was extracted from 1 mL whole blood of each participant and subjected to gel electrophoresis and β-globin PCR for the assessment of DNA quality and integrity. All samples showed compact and brighter bands on 1% agarose gel showing high quality of DNA (Additional file 1: Figure S1A). The β-globin amplification products were also observed in all DNA samples showing that DNA is intact and suitable for any downstream application (Additional file 1: Figure S1B). Cross reactivity of primers for *LT antigens* of both viruses were evaluated among BKPyV and JCPyV. Primer set of BKPyV *LT antigen* as well as JCPyV *LT antigen* amplified only their respective targets in each virus showing the specificity of primers for BKPyV and JCPyV, respectively. No cross reactivity was observed between the primers of BKPyV and JCPyV (Additional file 2: Figure S2).

### Real-time PCR assay for the quantification of BKPyV and JCPyV viral loads

**Fig. 1:**
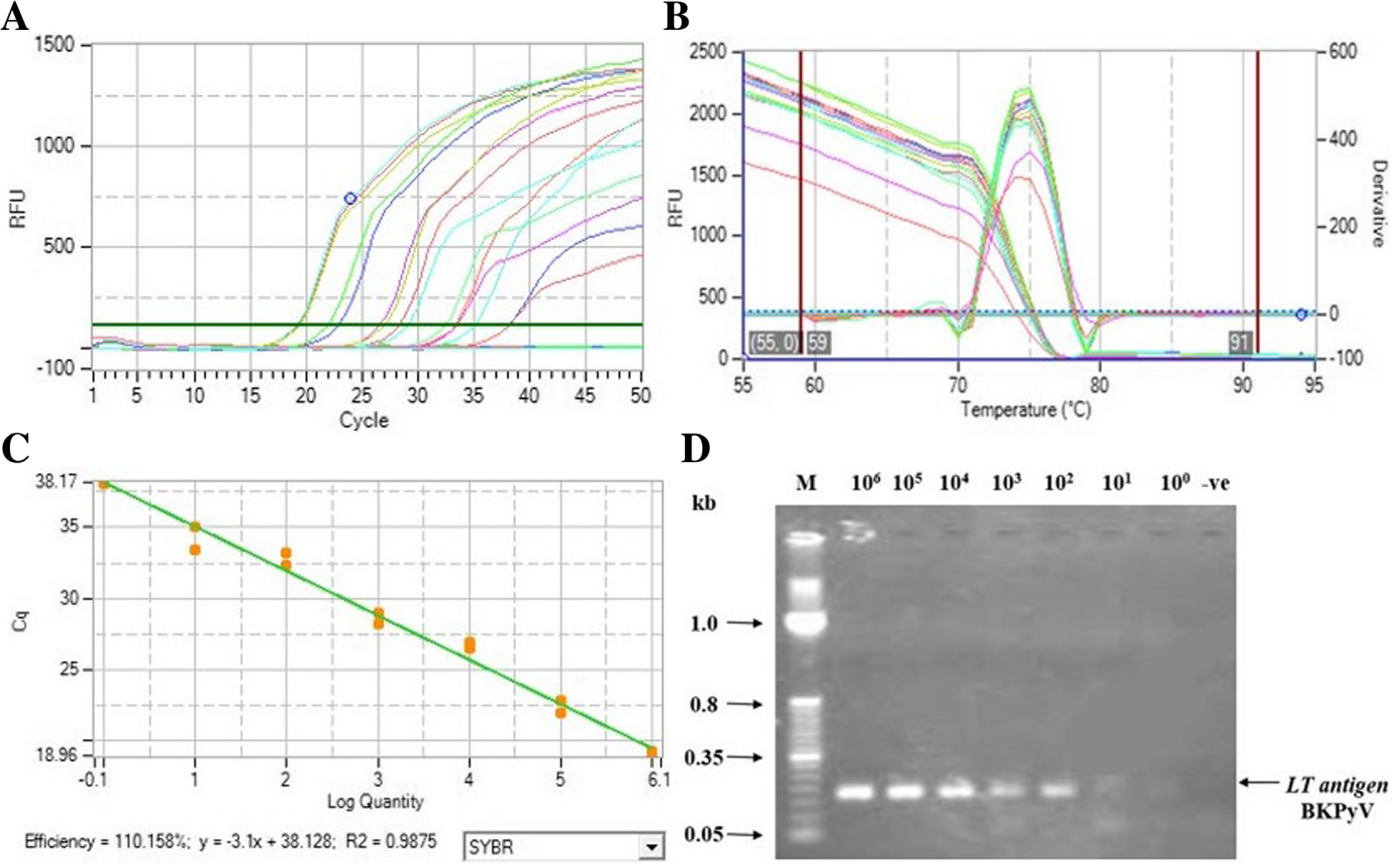
Amplification and standard curves for BKPyV dilutions. A standard curve was generated using duplicate ten-fold serial dilutions of purified *LT*-BKPyV-pcDNA3 template. The linear range was set to lie between 10^6^ to 10^0^ copies/reaction. The assay efficiency was checked by (**a**) amplification plots of BKPyV standards dilution (**b**) Melt curves analysis for BKPyV target gene with a peak at 74 °C (**c**) standard curve analysis for BKPyV dilutions. The assay efficiency was calculated 110% with R2 = 0.98. **d** The amplified product of each dilution was analyzed on agarose gel. Lane 1: molecular mass marker; Lane 2–8: dilution (copy number 10^6^ to 10^0^); Lane 9: negative control

**Fig. 2:**
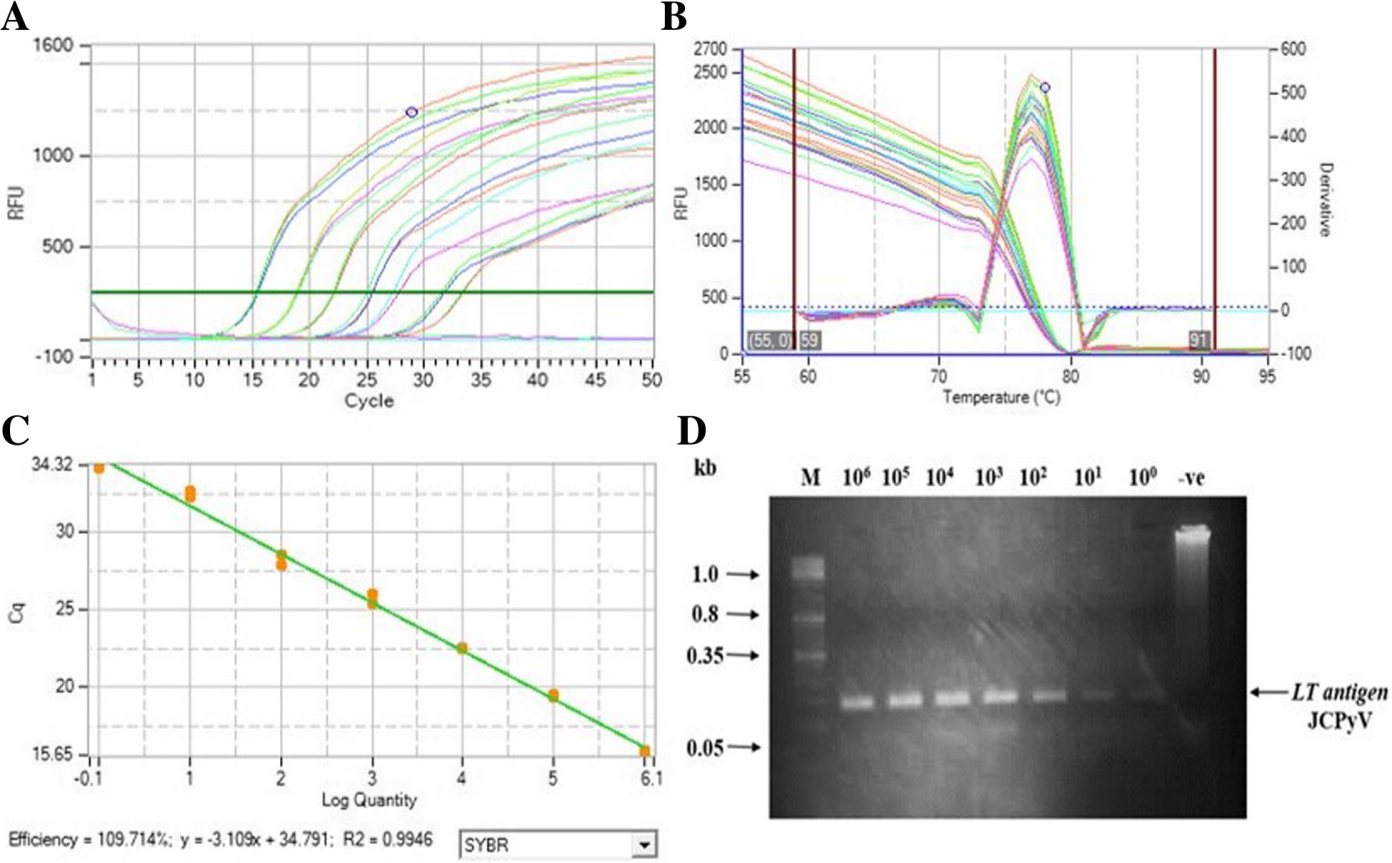
Amplification and standard curves for JCPyV dilutions. A standard curve was generated using duplicate ten-fold serial dilutions of purified *LT*-JCPyV-pcDNA3 template. The linear range was set to lie between 10^6^ to 10^0^ copies/reaction. The assay efficiency was checked by (**a**) amplification plots of JCPyV standards dilution (**b**) Melt curves analysis for JCPyV target gene with a peak at 77 °C (**c**) standard curve analysis for JCPyV dilutions. The assay efficiency was calculated 109% with R2 = 0.99. **d** The amplified product of each dilution was analyzed on agarose gel. Lane 1: molecular mass marker; Lane 2–8: dilution (copy number 10^6^ to 10^0^); Lane 9: negative control

For detection and quantification of BKPyV and JCPyV viral load in blood samples by qPCR, standard curves were plotted for each virus by using 10 fold serial dilutions of *LT*-BKPyV-pcDNA3 and *LT*-JCPyV-pcDNA3 plasmids. The concentrations of these positive standards dilutions were adjusted between 10^6^ and 10^0^ copies. The Ct values for BKPyV positive control dilutions were in the range of 17–37 (Fig. 1a) while 15–34 for JCPyV positive control dilutions (Fig. 2a). Results of standard curves were further analyzed with PikoReal 2.2 software. Melt curves analysis manifested peak at 74 and 77 °C for BKPyV and JCPyV target genes respectively (Figs. 1b and 2b). The assay efficiency was calculated 110% with R2 = 0.98 for *LT*-BKPyV-pcDNA3 (Fig. 1c) while 109% with R2 = 0.99 for *LT*-JCPyV-pcDNA3 (Fig. 2c). The PCR product of each dilution was analyzed on agarose gel and bands of respective molecular weight with decreasing intensities from high to low dilution were observed for both BKPyV (Fig. 1d) and JCPyV (Fig. 2d).

**Fig. 3:**
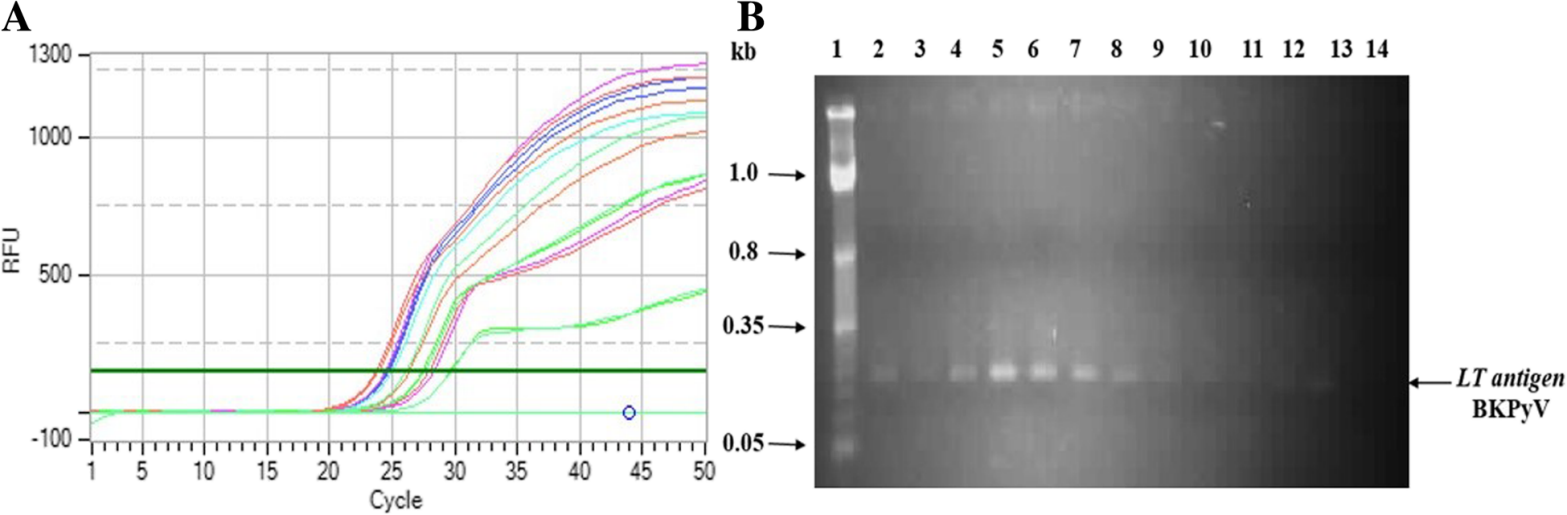
Real time assay for the quantification of BKPyV in healthy blood samples. The qPCR was performed by using 30 ng DNA of each sample and virus copy number was determined by comparing with standard curve of known BKPyV dilutions. **a** Amplification plots of some representative positive samples for BKPyV. **b** The amplified product of these representative positive samples was analyzed by gel electrophoresis. The amplified product showed band of 151 bps corresponding to BKPyV *LT*. Lane 1: molecular mass marker; Lane 2–13: representative positive samples for BKPyV; Lane 14: negative control

**Fig. 4:**
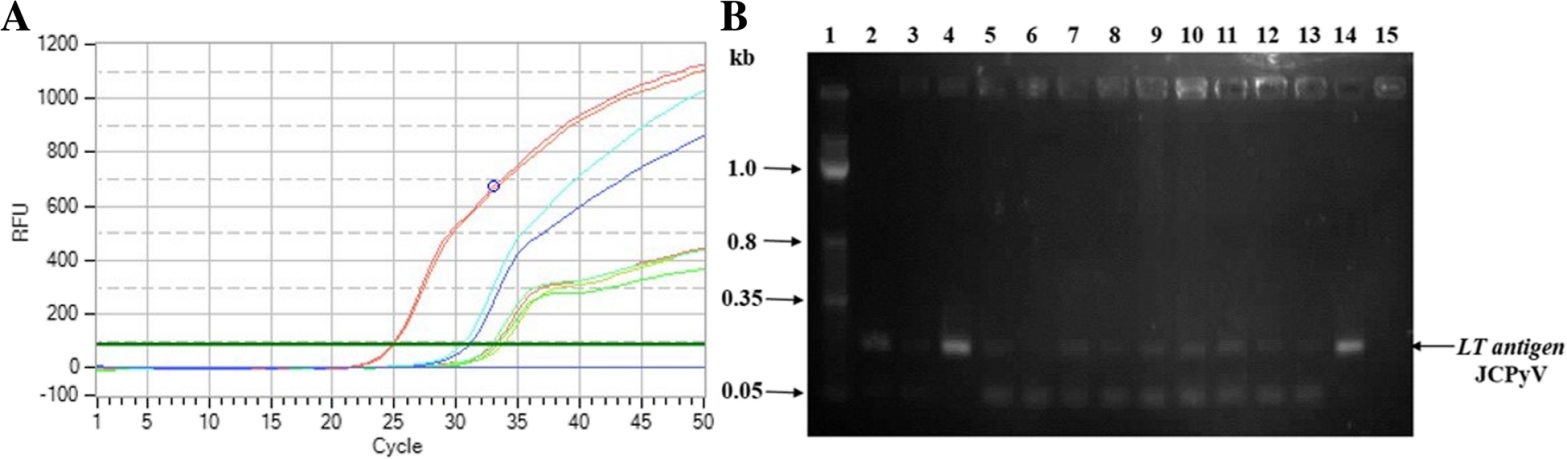
Real time assay for the quantification of JCPyV in healthy blood samples. The qPCR was performed by using 30 ng DNA of each sample and virus copy number was determined by comparing with standard curve of known JCPyV dilutions. **a** Amplification plots of some representative positive samples for JCPyV. **b** The amplified product of these representative positive samples was analyzed by gel electrophoresis. The amplified product showed band of 177 bps corresponding to JCV *LT*. Lane 1: molecular mass marker; Lane 2–14: representative positive samples for JCPyV; Lane 15: negative control

The 30 ng of DNA isolated from each blood sample was used in real time PCR reaction for the quantification of BKPyV and JCPyV. The positive samples showed comparable amplifications of *LT antigen* of BKPyV and JCPyV. Amplification cycles (Ct values) of positive samples were within range of standard curve Ct values of both BKPyV (Fig. 3a) and JCPyV (Fig. 4a). Notably, melt curves of positive samples were also specific for target genes (data not shown). The BKPyV and JCPyV positive samples with low Ct values were also positive on 2% agarose gel but samples with high Ct values did not appear in the agarose gel (Figs. 3b and 4b). Viral load in each positive sample was calculated by comparing its Ct value with Ct values of standard curve.

### The prevalence of BKPyV and JCPyV in different age groups of Pakistani Population

**Fig. 5:**
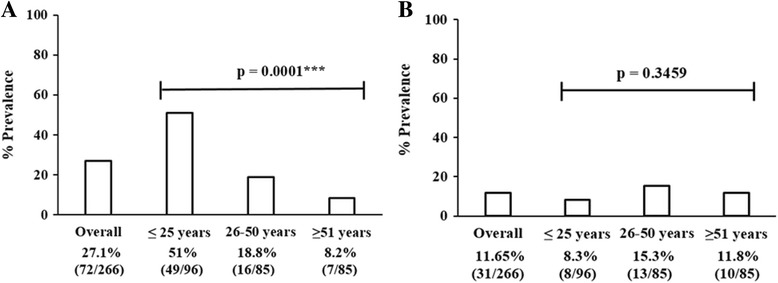
The prevalence of BKPyV and JCPyV in the blood of healthy Pakistani population. Overall 266 blood samples from healthy individuals of Pakistan were analyzed for the presence of BKPyV and JCPyV. **a** The bar graph shows the overall and age-wise prevalence of BKPyV in the studied population. **b** The bar graph shows the overall and age wise prevalence of JCPyV in the studied population

Overall, the BKPyV DNA was detected in 27.1% (72/266) individuals. The BKPyV prevalence was 51% (49/96), 18.8% (16/85), and 8.2% (7/85) in young, middle and elder age groups respectively, showing that the presence of BKPyV significantly varies (*p*-value = 0.0001) in each age group and BKPyV positivity ratio decreases with the increase in age (Fig. 5a). On the other hand, the JCPyV DNA was detected in 11.6% (31/266) of studied population. Contrary to BKPyV, JCPyV prevalence was slightly higher in middle and elder age groups in comparison to the young age group. JCPyV positivity was 8.3% (8/96), 15.3% (13/85), and 11.8% (10/85) in young, middle and elder age groups respectively. The difference in term of JCPyV positive samples was statistically not significant (*p*-value = 0.345) among different age groups (Fig. 5b).

### The BKPyV and JCPyV viral loads in individuals of different age groups from Pakistan

**Fig. 6:**
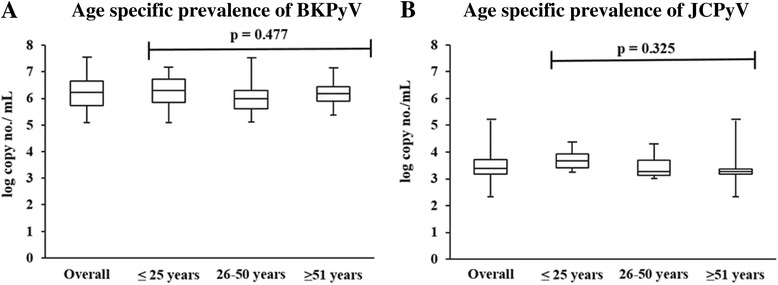
The age specific prevalence and copy number of BKPyV and JCPyV in the blood of healthy Pakistani population. Overall 266 blood samples from healthy individuals of Pakistan were analyzed for the presence of BKPyV and JCPyV. The virus copy numbers were also calculated for positive samples. The virus load in the positive samples was expressed as log copy number/mL. **a** Box-whisker plots show the BKPyV positive samples and range of virus copy number in the positive samples of overall and three age groups (≤25 years, 26–50 years and ≥ 51 years). **b** Box-whisker plots show the JCPyV positive samples and range of virus copy number in the positive samples of overall and three age groups (≤25 years, 26–50 years and ≥ 51 years)

BKPyV and JCPyV viral copy numbers in the positive samples were also calculated by comparing the Ct values with Ct values of standard curve and expressed as log copy number per mL. Age specific viral load was monitored and represented in the Box-whisker plots. Overall, the BKPyV viral load was within 5.08–7.5 log copies per mL of blood. Median log copies of BKPyV for young age group (≤25 years) was 6.3 log copies/mL of blood while for middle and elder age group (26–50 years and ≥ 51 years) it was 5.9 log and 6.1 log copies per mL of blood. These results demonstrate that BKPyV viral load is (*p*-value = 0.477) (Fig. 6a). The JCPyV viral load was within 2.3–5.1 log copies per mL of blood. Viral load was maximum in eldest age group (≥51 years) with median 3.6 log copies per mL of blood_._ In other two age groups; ≤ 25 years and 26–50 years, median copy number was similar 3.2 log. Statistically, the JCPyV viral load does not differ in studied age groups (*P*-Value = 0.325) (Fig. 6b). The prevalence and viral loads of BKPyV and JCPyV in all age groups along with mean and median are mentioned in Table 2

**Table 2:** Age specific viral status in blood samples evaluated for BKPyV and JCPyV

Age Groups (Years)	BKPyV			JCPyV			BKPyV & JCPyV Co-infection
	Positive sample % (n)	Viral copy no. (log/mL of blood)	Mean ± S.D	Positive sample % (n)	Viral copy no. (log/mL of blood)	Mean ± S.D	Positive sample % (n)
≤25 years	51.0% (49/96)	5.08–7.14	6.24 ± 0.56	8.3% (8/96)	3.25–4.35	3.70 ± 0.37	0.7% (2/266)
26–50 years	18.8% (16/85)	5.10–7.5	6.04 ± 0.63	15.3% (13/85)	3.02–4.28	3.44 ± 0.44	
≥51 years	8.2% (7/85)	5.37–7.15	6.19 ± 0.56	11.8% (10/85)	2.34–5.17	3.32 ± 0.82	
Overall	27.1% (72/266)	5.08–7.5	6.19 ± 0.57	11.65% (31/266)	2.34–5.17	3.47 ± 0.52	

.

### Overall comparison between BKPyV and JCPyV in term of prevalence and viral load

**Fig. 7:**
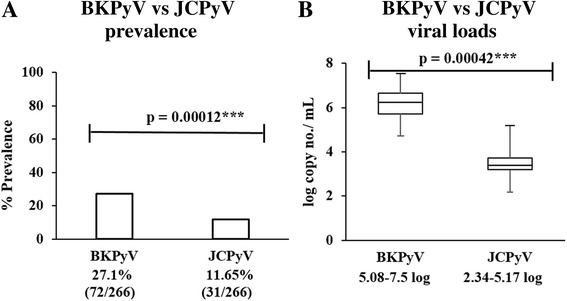
A comparison of the prevalence as well as viral between BKPyV and JCPyV in the blood of healthy Pakistani population. Overall 266 blood samples from healthy individuals of Pakistan were analyzed for the presence of BKPyV and JCPyV. **a** The bar graph shows the overall difference among the prevalence of BKPyV and JCPyV in the studied population. **b** Box-whisker plots compares the BKPyV and JCPyV range of virus copy number in the positive samples of overall population

Altogether, prevalence of BKPyV was compared with JCPyV in the studied population. It was concluded that BKPyV prevalence (27.1%) was significantly (*p*-value = 0.00012) high in comparison to JCPyV (11.6%) in Pakistani population (Fig. 7a). Similarly, BKPyV and JCPyV viral load was compared and found that BKPyV viral load (5.08–7.5 log copies per mL of the blood) was significantly higher than JCPyV load (2.34–5.17 log copies per mL of blood) (*p*-value = 0.00042) (Fig. 7b).

## Discussion

Human polyomaviruses are circular double stranded DNA viruses which belong to *Polyomaviridae* family of viruses [25]. Out of 13 human polyomaviruses, only BKPyV, JCPyV, MCPyV and TSPyV have been directly linked with clinical ailments [30]. We particularly focused on BKPyV and JCPyV due to their association with specific human diseases: polyomavirus associated nephropathy and progressive multifocal leukoencephalopathy respectively [31]. The frequencies of these viruses vary in different geographical regions and population, in different biological samples and even within a same population due to the choice of technique used for the detection [32–34]. In Pakistan, unfortunately not even a single report, to the best of our knowledge is available about the prevalence of these hazardous viruses. Our study being first ever report on the distribution of polyomaviruses in Pakistan not only describes the prevalence of these viruses but also quantified viral load in positive samples. A total of 266 apparently healthy individuals were recruited in this study and these individuals were divided in three age groups; ≤ 25 years, 26–50 years and ≥ 51 years. The DNA was extracted from whole peripheral blood and used for the detection and quantification of BKPyV and JCPyV by real time PCR. The results described the overall prevalence of BKPyV and JCPyV as 27.1 and 11.6% respectively. The overall viral load was in the range of 5.08–7.5 log and 2.34–5.17 log for BKPyV and JCPyV respectively.

Previously, the seroprevalence of these viruses was studied mainly and BKPyV prevalence was reported to be 55–85% [35] and JCPyV to be 50–80% [36]. Likewise, molecular detection of these viruses in both immunosuppressed and immunocompetent individuals has also been reported in some studies where serum/plasma or urine samples were targeted for BKPyV and JCPyV detection. These studies reported the urinary shedding of BKPyV and JCPyV in healthy population 7 and 19% respectively [37]. Discordant data regarding the prevalence of these viruses in healthy individuals has previously been published. By and large, blood polyomavirus positivity has varied from 0 to 90% in healthy immunocompetent individuals [17]. Similarly, different studies have described variable range of BKPyV and JCPyV viral loads in healthy individuals from different parts of the world [18, 37–40]. These viruses were less frequent overall in urine and/or serum/plasma of healthy individuals [17]. The low viral load in these body fluids could be explained by the fact that both of these viruses remain latent in healthy population and viruria or viremia increase only as a consequence of their reactivation [20]. In contrast to most of the previous studies, our protocol uses DNA extracted from peripheral whole blood instead of serum or plasma. This modification in already established serum/plasma based protocols not only simplifies the procedure but is also of great importance because recent reports suggest that PBMC, leukocytes and lymphocytes may serve as a site of latency for these viruses in healthy population [17, 41]. Thus, future studies aiming to verify this hypothesis may benefit from our suggested method.

Previous studies have reported variable molecular prevalence rates in different populations. In Italy, for instance, the molecular prevalence of BKPyV was reported 22% in lymphocytes which is comparable to our results that is 27% (72/266) overall prevalence [17]. While in a cohort of Chinese population 42.1% BKPyV prevalence in peripheral blood leukocytes was reported [19]. In our results, the frequency of BKPyV was maximum in the youngest age group (≤25 years) 51% (49/96) and decreased to 8.2% (7/85) in the eldest age group (≥51 year). Contradicting studies are available regarding age-wise prevalence of BKPyV. A study on age specific molecular prevalence of BKPyV in Germany showed that increasing age has no significant effect on the molecular prevalence of polyomavirus in peripheral blood [18]. However, a study targeting European population reported seroprevalence rates going down from 80% in young adults to 55% in older age group [42]. These results appear to agree with our observation. Other studies have also reported similar trends in age specific seroprevalence studies of BKPyV [20, 43, 44].

In the present study JCPyV DNA was detected only in 11.6% (31/266) participant that is comparable with earlier reports from various Asian populations, for instance 8% in healthy Chinese population and 14% in healthy tribal Indian population [17, 38]. A peculiar age group specific trend in JCPyV infection, different from BKPyV, was observed. Rate of positivity for JCPyV first increased from young to middle age group before declining again in older age group. The positivity rate in old age group though less than middle age group, nonetheless remained higher as compared to young age group. In the youngest age group (≤25 years), the frequency of JCPyV was 8.3% (8/96) which reached up to maximum in middle age groups (26–50 years) to 15.3% (13/85), and then in eldest age groups (≥51 years) decreased to,11.8% (10/85). However, this difference in JCPyV frequency was not significant statistically (*p* = 0.345). Contradictory findings have been reported previously from different parts of the world regarding the age group specific prevalence of JCPyV. For example, Delbue et al. found that age has no significant effect on the molecular prevalence of polyomaviruses [18]. Contrary to that, Sadeghi et al. reported that seroprevalence of JCPyV in Australian population increases from 60 to 68% in middle-ages and then decrease to 64% in oldest ages [45]. Although a similar trend was noticed in our study, rate of JCPyV infection in our target population was much lower than the Australian cohort.

We also quantified the viral loads in the positive samples and expressed as log copies per mL of blood. The viral loads calculated in our study were in the range of 5–7.5 and 2.34–5.17 log copies per mL of blood for BKPyV and JCPyV respectively, without any significant difference in each age group. The high viral load calculated in our study could be due to the use of whole blood instead of serum/plasma because whole blood contains reservoirs of latent BKPyV and JCPyV. This hypothesis is supported by Delbue et al.’s study where the researchers reported high viral load of both BKPyV (7 × 10^3^ copies/μg) and JCPyV (2 × 10^4^ copies/μg) using whole peripheral blood of immunocompetent individuals similar to the present report [18]. The viral load in this study was described as “copies/μg” which becomes equivalent to our study once converted into log copies per ml.

The prevalence of BKPyV and JCPyV in the immunocompetent individuals in our study is higher than Europe and USA but it is less than already reported in Asian countries such as China and India. For example, Gu et al. reported overall prevalence of BKPyV DNA in Peripheral blood leukocytes (PBLs) of healthy adult individuals in China as 42.1% [19], which is considerably higher than the present report. Similarly, viral load described in our study is lower than already described in India. Chattaraj et al. reported high viral load of JCPyV (7.77 × 10^5^ copies/ml) in the blood of healthy individuals from Indian tribes [38], higher than our report. Based on the very few reports that are available from Asia, it may be hypothesized that incidence and viral load of these ployomaviruses in this part of the world is higher than other regions. However, detailed studies may be required to substantiate this claim.

It is well established that nutritional deficiency might result in immunodeficiency which in turn can augment the receptiveness towards different infections [46]. The majority of population in both India and Pakistan cannot meet their dietary requirements due to poor socio economical conditions. The nutritional deficiencies could also explain the high viral load particularly in this part of the world. However, more studies will be needed to understand the contribution of nutritional deficiencies in immunosuppression among South Asian population.

In comparison to the JCPyV in our findings, BKPyV had higher viral load and molecular prevalence in healthy population of Pakistan. These significant differences in the prevalence and viral load of these viruses can be explained due to their specific virus-host interaction and pathogenesis. We also investigated the probability of co-infection with both of the species and found that only 0.7% of the individuals included in our study had a co-infection of BKPyV and JCPyV. Similar co-infection rates (1%) have previously been reported by Egli et al. in Switzerland [43].

In this study, we successfully demonstrated detection of both JCPyV and BKPyV species in peripheral blood samples of apparently healthy Pakistani individuals, therefore our study highlights the utility of blood as suitable sample type for polyomavirus surveillance studies. Moreover, sensitive and specific molecular detection method used in our study also lends credence to the hypothesis that PBMCs can serve as sites of latency for polyomaviruses. However, due to the unavailability of urine samples in sufficient numbers and quality suitable for molecular detection of virus, this important analysis could not be included in present study. Nonetheless, our report has provided baseline data upon which further studies can be designed for better estimation of BKPyV and JCPyV prevalence in general Pakistani population.

## Conclusion

The present study provides an important baseline data on prevalence and viral load of BKPyV and JCPyV in the blood of apparently healthy Pakistan population. The BKPyV prevalence 27.1% (72/266) was higher than JCPyV 11.6% (31/266). The prevalence of BKPyV significantly decreased with increase in age while JCPyV positivity rate slightly increased with increasing age. Likewise, the viral load of BKPyV was significantly higher than JCPyV. However, both BKPyV and JCPyV viral load was not correlated with the individual ages. Conclusively, this study on polyomaviruses in Pakistan manifested the existence of both BKPyV and JCPyV in the blood of apparently healthy Pakistani population.

## Additional files

Additional file 1: Figure S1. Analysis of genomic DNA (gDNA). A) Analysis by agarose gel. The genomic DNA was isolated from the whole blood and loaded on 1% agarose gel. The compact and brighter bands indicate the intact gDNA. Lane 1: molecular mass marker; Lane 2–10 representative genomic DNA from blood samples. B). Quality assay by β-globin amplification. Lane 1: molecular mass marker; Lane 2: Negative control; Lane 3–10; β-globin amplified product from representative genomic DNA. (JPG 66 kb)

Additional file 2: Figure S2. Cross reactivity between BKPyV and JCPyV primers. The BKPyV and JCPyV primers specificity was checked by PCR using BKPyV and JCPyV templates for each primer set. All primers were specific having no cross reactivity with other viruses. Lane 1: molecular mass marker; Lane 2,3: *LT*-BKPyV-pcDNA3 template with BKPyV and JCPyV *LT antigen* primers, respectively; Lane 6,7: *LT*-JCPyV-pcDNA3 template with BKPyV and JCPyV *LT antigen* primers, respectively; Lane 4, 8: non template control pcDNA3 for BKPyV and JCPyV *LT antigen* primers, respectively; Lane 5, 9: negative control (water) for BKPyV and JCPyV *LT antigen* primers, respectively. (JPG 46 kb)

## Abbreviations

BKPyV: BK polyomavirus
JCPyV: JC polyomavirus
LT antigen: Large tumor antigen
NCCR: Non coding control region
PBMCs: Peripheral blood mononuclear cells
PyV: Polyomavirus
qPCR: Real time PCR
ST antigen: Small tumor antigen
VP: Viral capsid protein
