# Supplementary figures and images for: Specific and quantitative detection of Human polyomaviruses BKPyV and JCPyV in the healthy Pakistani population
Source: Virol J. 2017 Apr 24;14:86. doi: 10.1186/s12985-017-0752-2 (PMC5404684; doi:10.1186/s12985-017-0752-2)

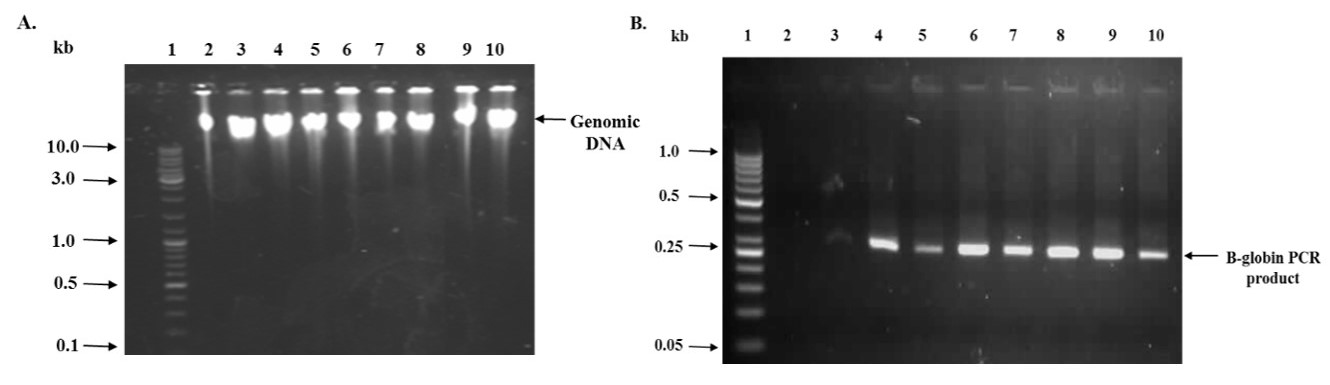

Supplement: Supplementary file 1 — Analysis of genomic DNA (gDNA). A) Analysis by agarose gel. The genomic DNA was isolated from the whole blood and loaded on 1% agarose gel. The compact and brighter bands indicate the intact gDNA. Lane 1: molecular mass marker; Lane 2–10 representative genomic DNA from blood samples. B). Quality assay by β-globin amplification. Lane 1: molecular mass marker; Lane 2: Negative control; Lane 3–10; β-globin amplified product from representative genomic DNA. (JPG 66 kb) [file 12985_2017_752_MOESM1_ESM.jpg]

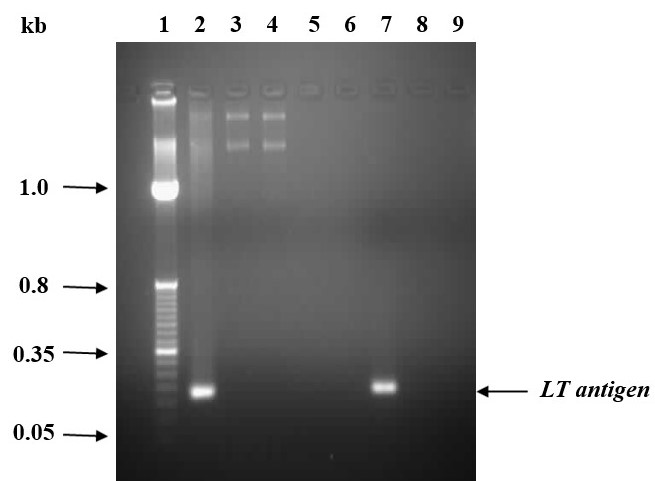

Supplement: Supplementary file 2 — Cross reactivity between BKPyV and JCPyV primers. The BKPyV and JCPyV primers specificity was checked by PCR using BKPyV and JCPyV templates for each primer set. All primers were specific having no cross reactivity with other viruses. Lane 1: molecular mass marker; Lane 2,3: LT-BKPyV-pcDNA3 template with BKPyV and JCPyV LT antigen primers, respectively; Lane 6,7: LT-JCPyV-pcDNA3 template with BKPyV and JCPyV LT antigen primers, respectively; Lane 4, 8: non template control pcDNA3 for BKPyV and JCPyV LT antigen primers, respectively; Lane 5, 9: negative control (water) for BKPyV and JCPyV LT antigen primers, respectively. (JPG 46 kb) [file 12985_2017_752_MOESM2_ESM.jpg]
